# Influence of Contour Scan Variation on Surface, Bulk and Mechanical Properties of LPBF-Processed AlSi7Mg0.6

**DOI:** 10.3390/ma16083169

**Published:** 2023-04-17

**Authors:** Theresa Buchenau, Marc Amkreutz, Hauke Bruening, Bernd Mayer

**Affiliations:** 1 Fraunhofer Institute for Manufacturing Technology and Advanced Materials, Wiener Straße 12, 28359 Bremen, Germany; marc.amkreutz@ifam.fraunhofer.de (M.A.); hauke.bruening@ifam.fraunhofer.de (H.B.); bernd.mayer@ifam.fraunhofer.de (B.M.); 2 Faculty of Production Engineering, University of Bremen, 28359 Bremen, Germany

**Keywords:** additive manufacturing, laser powder bed fusion, LPBF, PBF-LB, contour scan variation, mechanical testing, tensile strength, fatigue, AlSi7Mg0.6, surface quality, bulk quality, areal surface texture parameters

## Abstract

Metal additive manufacturing technologies have great potential for future use in load-bearing aerospace applications, requiring a deeper understanding of mechanical performance and influencing factors. The objective of this study was to investigate the influence of contour scan variation on surface quality, tensile and fatigue strength for laser powder bed fusion samples made of AlSi7Mg0.6 material and to create high-quality as-built surfaces. The samples were produced with identical bulk and different contour scan parameters to accommodate the investigation of the impact of as-built surface texture on mechanical properties. The bulk quality was evaluated by density measurements according to Archimedes’ principle and tensile testing. The surfaces were investigated using the optical fringe projection method, and surface quality was assessed by the areal surface texture parameters Sa (arithmetic mean height) and Sk (core height, derived from material ratio curve). Fatigue life was tested at different load levels, and the endurance limit was estimated based on a logarithmic-linear relation between number of cycles and stress. All samples were found to have a relative density of more than 99%. Surface conditions distinctive in Sa and Sk were successfully created. The resulting mean values of the ultimate tensile strength $\sigma_{ult}$ are between 375 and 405 MPa for 7 different surface conditions. It was confirmed that the influence of contour scan variation on bulk quality is insignificant for the assessed samples. Regarding fatigue, one as-built condition was found to perform as well as surface post-processed parts and better than the as-cast material (compared to literature values). The fatigue strength at the endurance limit for $10^{6}$ cycles is between 45 and 84 MPa for the three considered surface conditions.

## 1. Introduction

Additive manufacturing (AM) technologies, in particular laser powder bed fusion (LPBF), are of extraordinary interest to the aerospace industry. Advantages of these technologies include a large increase in geometrical freedom and potential savings of material and overall production cost [1,2,3,4].

It is also desirable to use AM technology in load-bearing applications, but standards for part certification and quality assurance are not yet established. Hence, there is currently still a restriction to non-critical parts in aerospace systems [5,6]. Part of the work done to gain an understanding of the process–material–property relations needed as a foundation for part qualification is summarized in Section 1.1. Section 1.2 explains the contribution of this work to that same understanding.

### 1.1. Mechanical Properties of LPBF-Processed AlSi Alloys

Different review papers have suggested that there is an extensive number of studies on LPBF processing of materials like Ti-64 [7,8,9,10,11], Inconel 718 [11,12,13,14] or 316L steel [15,16,17,18,19]. LPBF-processing of aluminium alloys, however, has only gained importance in recent years [20,21,22,23,24,25]. Aboulkhair et al. found that this is related to the particularly challenging properties of aluminium alloys and aluminium alloy powders for laser processing. The powders are generally characterized by low flowability, which impacts powder layer recoating, and are prone to oxidation, causing porosities. Moreover, the high reflectivity of the common LPBF process wavelength range, low laser absorption and high thermal conductivity result in a need for high laser power [21].

Nonetheless, LPBF processing of aluminium alloys is interesting, especially for lightweight construction applications, as they are lightweight, strong, corrosion-resistant and highly weldable. Combined with the geometrical freedom enabled by LPBF processing, they are suitable for tailoring parts for numerous purposes within automotive, aerospace and other industries [21].

The best LPBF-processable alloys are aluminium–silicon-based, and the most commonly-investigated one is AlSi10Mg. The silicon phase in the solidified LPBF material contributes to limit crack initiation and propagation due to the LPBF-typical fine microstructure and improves its tensile strength as compared to the cast material [4,24,26].

In particular, the alloys AlSi10Mg, AlSi12 and AlSi7Mg are considered ‘highly printable’ [24]. For these materials, ultimate tensile strength (UTS) values between 300 and 450 MPa in as-built condition are reported [4,24,27,28,29,30,31,32,33,34,35,36].

Many publications assess the effect of heat treatment [30,31,33,36,37,38,39,40,41], and there is some work addressing the effect of surface post-processing [42,43,44,45] or positioning on the build platform [39,46] on mechanical properties.

In this section, an overview of recent work on mechanical properties is given. The focus is on investigations on tensile and fatigue behavior of LPBF-processed aluminium alloys, particularly the AlSi7Mg0.6 alloy.

#### 1.1.1. Tensile Properties

Yang et al. investigated the effect of heat treatments on microstructure and mechanical behavior anisotropy for the AlSi7Mg0.6 alloy. They observed the typical LPBF fine microstructure in as-built condition due to the material’s fast cooling rate and a resulting higher strength than the as-cast alloy. Of the heat-treated samples, directly aged (T5) samples showed the highest strength and stress-relieved samples showed the largest elongation at fracture [31].

Similarly, Rao et al. found better tensile strength in as-built LPBF compared to the as-cast condition and observed that stress relaxation had a negative effect on yield strength (YS) and UTS while causing a slight improvement in ductility. A short solution heat treatment improved ductile behavior, and a longer treatment led to a decrease in YS and ductility [30].

Pereira et al. compared microstructure and mechanical properties of AlSi7Mg0.6 from LPBF and investment casting. They found that mechanical properties of LPBF can exceed aerospace qualification requirements for heat treated (T6) investment casting parts. They used direct aging heat treatment to improve ductility and hardness of LPBF-processed samples while maintaining a similar tensile strength as compared to as-built samples (e.g., mean UTS (as-built, vertical) of 435 MPa, after heat treatment 431 MPa) [33].

Zhang et al. looked into the effect of heat treatment for Er-containing AlSi7Mg0.6 and found that tensile properties are superior to the non-Er-containing alloy. The applied heat treatments improved ductility from 8% up to 19% for stress-relieved samples (with reduced tensile strength). Direct aging and T6 heat treatment both resulted in increased YS [36].

Advantages and disadvantages of different heat treatments compared to as-built ones were discussed by Mauduit et al. Amongst others, they found that the investigated heat treatments soft annealing and T6 resulted in isotropic mechanical properties. Soft annealing reduced tensile strength but removed residual stresses, artificial aging created the best UTS, but samples exhibited anisotropic mechanical properties. As-built samples already reached good mechanical properties but showed anisotropy. However, not applying heat treatment led to shorter production time and was less expensive [40].

Menezes et al. evaluated the effect of orientation on the build plate for as-built and heat-treated samples. Both conditions showed anisotropic behavior, where vertical specimens had lower YS and higher UTS. Comparing artificially aged and as-built samples, the latter showed lower YS [47].

Next to vertically (90°) and horizontally built (0°) samples, Denti included specimens built at a 45° angle in their investigation and observed a (slight) tendency for increasing tensile strength and decreasing elongation at fracture for steeper build angles [48].

In addition to heat treatment, Han et al. looked into the effect of laser surface remelting (LSR) for LPBF-processed AlSi10Mg and found that Ra (arithmetic mean of profile height variation) can be significantly improved by LSR. For as-built surfaces, they report an Ra>19 μm that improved to values below 1 µm for LSR-processed samples. In addition, LSR led to increasing micro-hardness. The applied heat treatment led to reduced tensile strength and improved ductility from 6% to 22% [41].

#### 1.1.2. Fatigue Properties

A full tension–tension loading Wöhler curve assessment with R=0 of the AlSi7Mg0.6 alloy using an endurance limit of $2\times 10^{6}$ cycles was performed by Bassoli et al. [49]. They obtained a result of 60±5.3 MPa and found that the alloy’s fatigue performance under the applied processing conditions was slightly lower but still comparable to reported literature values for the AlSi10Mg alloy [50]. Surface texture parameters were not specified, but they mentioned that the samples had not received any post-treatment.

Grande et al. [39] investigated the relationship of heat treatment and tensile strength as well as the effect of position on the build platform on fatigue life. They produced specimens with densities > 98.8% and as-built YS of 222 MPa and UTS of 417 MPa. They found that stress relief reduced tensile performance. Their fatigue results suggest that the position on the build platform does not have a significant influence on the endurance limit (at $10^{7}$ cycles: 127 MPa internal vs. 137 MPa external regions) of the heat-treated specimens. Fatigue samples were sandblasted to improve surface texture prior to fatigue testing.

Denti and Sola [43] looked into the effect of different post-processing technologies (e.g., sandblasting, plastic media blasting and laser shock processing) on axial fatigue. They found that the evaluated surface processing techniques improved the areal arithmetic mean surface height deviation Sa by up to 77%. The lowest Sa values were achieved by plastic media blasting. The peak stress level at the endurance limit of $2\times 10^{6}$ was improved by up to 80% with respect to the as-built $\sigma_{max}$ of 50 MPa. Fatigue performance was also improved by post-processing techniques not enhancing the surface quality, which led them to the conclusion that both the improvement of surface quality and the introduction of compressive residual stresses can play a role when looking at LPBF-processed aluminium alloy parts.

The impact of sample location on the build platform, orientation and variation between production batches was studied by Cacace et al. [46]. By analyzing mechanical property data of three batches with randomly allocated sample positions, they found that part position did not have an influence on tensile strength but did affect low cycle fatigue performance.

Nasab et al. [51] investigated the combined effect of volumetric and surface defects. They looked into as-built surfaces with different contour scans, trying to promote typical defects to show their effect on rotating bending fatigue. The defect depths were analyzed by optical line-of-sight measurement, as well as polished cross-sections. Material removal depths of up to 200 μm were suggested, depending on the surface condition. They state that contact and non-contact surface texture measurements cannot provide information on fatigue-critical surface features as comprehensively as investigations into polished cross-sections.

In previously published work, the authors of this paper evaluated crack initiation behavior and surface fatigue relations for AlSi7Mg0.6 for three different groups of as-built samples. We assessed the applicability of valley depth Sv and reduced valley depth Svk and found that Svk is especially useful when considering coarser as-built surfaces, since they tend to exhibit crack initiation from multiple surface defects [52].

### 1.2. Motivation and Objective

Most of the studies summarized deal with the influence of heat treatment and build direction (horizontal/vertical) on tensile properties. While tensile properties are an important starting point in understanding a material’s mechanical behavior and are certainly relevant for various applications, for many aerospace, automotive, biomedical or other industrial purposes, resistance to periodic loading is of interest. In regard to fatigue life, surface texture plays an important role [4,6].

The majority of studies including the effect of surface condition on fatigue performance of the LPBF-processed AlSi7Mg0.6 material, as well as other aluminium alloys and other typical LPBF powder materials (e.g., Ti-64, 316L steel or Inconel 718), evaluate the application of different surface post-processing strategies, e.g., [7,10,15,42,45,53,54,55].

However, especially when considering complex geometries or parts with inner surfaces that are difficult to access with post-processing tools, it is desirable to produce as-built surfaces (including near-surface regions) good enough to perform reasonably well under cyclic loading. In addition to accessibility issues, using as-built parts saves time and cost due to reducing processing steps, since extensive post-processing becomes unnecessary.

In this paper, the effect of the as-built surface condition on mechanical properties is discussed. The ultimate aim is to create high-quality as-built surfaces.

The first step is to create distinctive as-built surface conditions by varying contour scan parameters (Section 3.1). Afterwards, the effect of these variations on bulk quality, characterized by density (Section 3.2) and UTS (Section 4.1), is investigated. Finally, a first selection of fatigue results is presented, showing the influence of as-built surface condition on fatigue resistance at a load level of 0.5 $\sigma_{ult}$ and the endurance limit (Section 4.2).

## 2. Materials and Methods

### 2.1. Manufacturing

The evaluated samples were manufactured in an LPBF process on a Trumpf TruePrint 1000 from AlSi7Mg0.6 aluminium alloy powder. The powder composition along with mass fractions of alloying elements are shown in Table 1. Specifications of geometries and manufacturing settings are given subsequently.

#### 2.1.1. Sample Geometry

**Figure 1:**
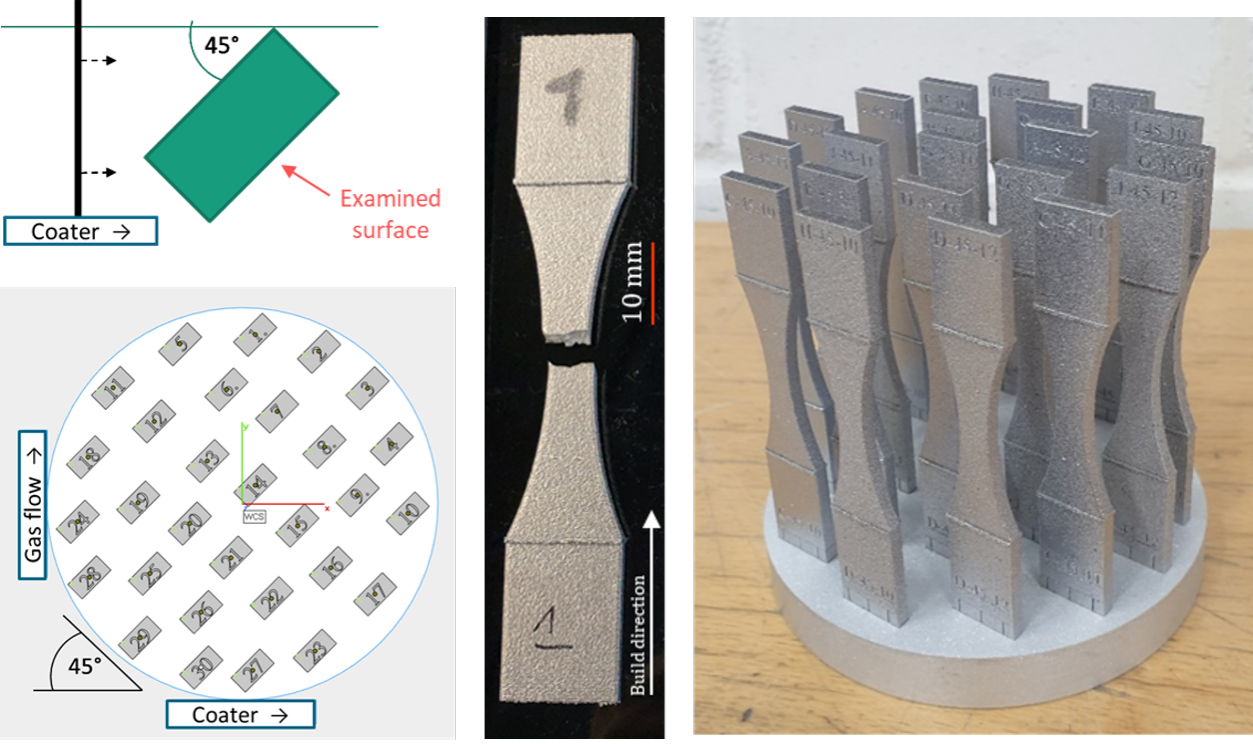
Top view: sample orientation with respect to coater (top left); example of build job layout (bottom left); individual tested sample (middle); finished build job on platform (right).

Two kinds of samples are used in this work: cuboids (height 10 mm, width 10 mm, thickness 5 mm) and fatigue specimens according to ASTM 466-15 [56] (height 80 mm, smallest cross section 6 mm, thickness 3 mm). The latter type is shown in Figure 1.

#### 2.1.2. Manufacturing Parameters

**Figure 2:**
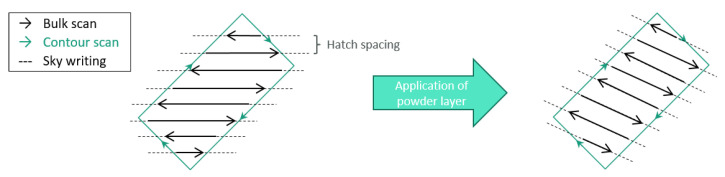
Exposure strategy for bulk and contour scan: bulk scan direction is rotated by 66° (schematic representation, not true to scale) after each powder layer application.

Detailed information on the manufacturing process is presented in Table 2 and Table 3 as well as Figure 1 and Figure 2.

Powder layers were exposed to the laser by a pattern of parallel lines in the bulk, changing direction by 66° after each layer, and a continuous scan of the geometric contour. Sky writing was applied to ensure the laser source was moving at the chosen speed prior to exposure.

The samples were placed on the build platform at a 45° angle with respect to the coater and gas flow, as shown in Figure 1. This angle was found to be most suitable regarding surface texture. In preliminary studies, comparable surface texture parameter values were found for both sides of the sample, supposedly because the effects of coater and gas flow compensate each other.

Bulk scan parameters were identical for all samples, as specified in Table 2, and originate from a previous density optimization study.

The contour scan parameters were varied, intending to achieve a variation of surface properties. Maintaining layer thickness, hatch distance and laser power, the scan speed was modified between 300 mm/s and 1800 mm/s, paired with the settings with and without additional pre-sinter at 50% laser power, resulting in a total of 10 manufacturing parameter combinations.

The samples with identical parameter combinations were named with a designated letter according to Table 4, with consecutive numbering; e.g., A1 → Contour parameter set A (scan speed 300 mm/s, with pre-sinter), mechanical testing sample No. 1.

### 2.2. Characterization and Testing

#### 2.2.1. Surface Texture

The surfaces were measured using a Keyence VR3200 fringe projection system. The micro camera setting at a magnification of 40× was applied, resulting in a lateral resolution of 7.4 µm. For the cuboid samples, selected ISO 25178 areal parameters were evaluated for a square area with an 8 mm length, measured perpendicular to the build direction on the side facing away from the coater, as indicated in Figure 1. A linear level operation, an S-filter of 20 µm and an L-filter of 0.25 mm were applied.

The chosen areal surface texture parameters to assess surface quality are Sa , the arithmetic mean height, and Sk , the core height from the material ratio curve. Sa was selected due to its common use in research and industry [57]. Sk is used because it gives more distinctive information on the surface texture (for details, refer to [58], p. 56).

The surface fatigue relation is shown using the material ratio curve parameter Svk , which is the reduced valley depth. The parameter was chosen because it describes the size of the valley population on the considered surface, rather than individual extreme values such as the maximum height Sz and the maximum valley depth Sv . More details can be found in [52]. Sa , Sk and Svk are defined in the ISO 25178-2 standard [59].

#### 2.2.2. Density

The first step toward the assessment of bulk quality was the measurement of part density. For this purpose, the cuboid samples were weighed in air and ethanol using the Mettler Toledo Delta Range XS603S precision balance. The density was calculated according to Archimedes’ principle as specified in ISO 3369 [60]. Each measurement was performed three times, and the final density result reported per sample is the respective mean value.

#### 2.2.3. Tensile Testing

The tensile strength was tested using a ZWICK/Z050 in accordance with ASTM E8M [61]. A preloading of 35 N and a speed setting of 0.48 mm/min were selected.

The required cross-sectional areas of the tested specimens were obtained from digital caliper measurements.

#### 2.2.4. Fatigue Testing

Fatigue life was tested on a DYNA-MESS 4S 20kN Z/D system at a frequency of 20 Hz and a stress ratio R=0.1 . The load levels were defined with respect to the mean value of the UTS for the tested surface conditions, $\sigma_{ult,mean}=\;392$ MPa. Corresponding values are specified in Table 5.

### 2.3. Workflow Summary

**Figure 3:**
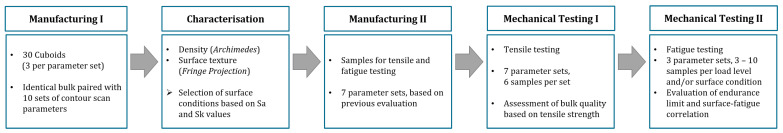
Workflow summary.

Figure 3 gives an overview of this work’s process steps.

At the first manufacturing stage, 30 cuboid samples were made. All of these were manufactured with identical bulk scan parameters, paired with 10 variations of contour scan parameters, resulting in 3 cuboid samples per parameter set combination.

Afterwards, the cuboids’ densities and surfaces were measured in order to get a first assessment of bulk quality and a rating based on the surface quality. Based on these evaluations, parameter sets were selected to produce samples for mechanical testing.

For seven manufacturing parameter sets chosen based on the cuboid assessment, six samples each were made for tensile testing. Tensile testing according ASTM E8M [61] was performed.

Finally, fatigue life was tested for a first selection of contour parameter sets, and their relationship with surface texture is discussed.

## 3. Results and Discussion of Preliminary Findings

The results presented in this section comprise density and surface texture characterization of the cuboid samples. The outcome is a selection of contour scan parameter sets for manufacturing the specimens for mechanical testing.

### 3.1. Surface Texture

#### 3.1.1. Visual Perception of Surface Quality

**Figure 4:**
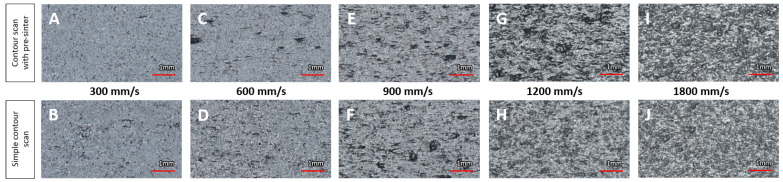
Microscopic images of samples with variation of contour scan speed, from lowest (left) to highest (right).

From visual inspection of the microscopic images in Figure 4, it can be observed that, at first sight, a variety of as-built surface conditions was achieved.

The A and B conditions look mostly smooth with small dots and few linear defects (length below 1 mm, oriented parallel to the layers). Increasing the contour scan speed, surfaces appear to have more and bulkier linear defects (C and D). The D image also seems a little blurry, which is a sign of increasing height variation on the surface. This effect becomes more clear when increasing scan speed even further (E and F). On surface F, there are a few circular shadows present, which may be spatter or local accumulations of powder particles. Surfaces G to J are hardly distinguishable visually. All show circular shadows of different sizes, which are mostly particle agglomerations and accumulations, and an underlying irregular structure. Surface G shows some darker areas, which may be an issue of different lighting conditions or height differences on the surface itself.

With increasing scan speed, the energy absorbed by the powder in the scanned path decreases. Due to the low energy, powder particles are only partially molten and attached to the surface, causing coarse surface quality.

#### 3.1.2. Selection of Contour Variation for Mechanical Testing from Surface Texture

**Figure 5:**
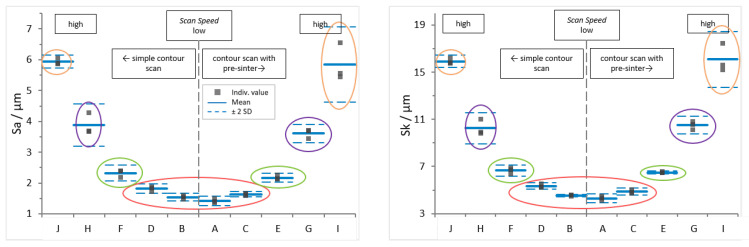
Sa and Sk for samples with different contour scan. L-filter 0.25 mm, S-filter 20 µm. Mean ± 2SD.

Figure 5 shows Sa (left) and Sk (right) values. The surface conditions are sorted by contour scan parameters. To the right, results for sample sets exposed to pre-sinter are presented, while to the left of each graph, results for simple contour scans are shown. The scan speed increases from the middle to the edge.

The graphs give the mean (blue line) ± two standard deviations (SD, dashed blue line). Colors mark the surface conditions that are distinctive per a 95% confidence interval (±2SD) applied to the parameter results for Sa and Sk . The first group (red) includes surface conditions A to D; conditions E and F form the second group (green); conditions G and H (purple) are the third group; and finally, the fourth group (orange) comprises conditions I and J.

A superficial look at the graphs presented in Figure 5 already confirms that the objective of creating surfaces with varying surface quality was met. This is also supported by the microscopic images in Figure 4. Numerical values are included in Table A1.

Based on the graphs, parameter sets to produce specimens for mechanical testing were selected.

From the smooth (red) group including surface conditions A to D, A was chosen as the set with the lowest mean values for Sa and Sk . C and D were selected to compare the possible impact of pre-sinter with otherwise identical process settings (see Table 4). G and H from the purple group are both considered for the same reason as conditions with higher parameter values.

Conditions E and F (green group) show comparable mean values for Sa and Sk , and it was decided to use set E, as it was the original starting parameter set of the contour variation study, and to discard condition F.

Parameter set J is chosen as the set with the highest mean value for Sa . Condition I, as the second coarse texture set (orange group), has a larger SD for both considered surface texture parameters and was discarded.

In summary, the following contour parameter sets are applied to produce the specimens for mechanical testing:

- Smooth surface parameter sets A, C and D (red group),
- Original parameter set E (green group),
- Coarse (purple group) and very coarse (orange group) parameter sets G, H and J.

### 3.2. Density

**Figure 6:**
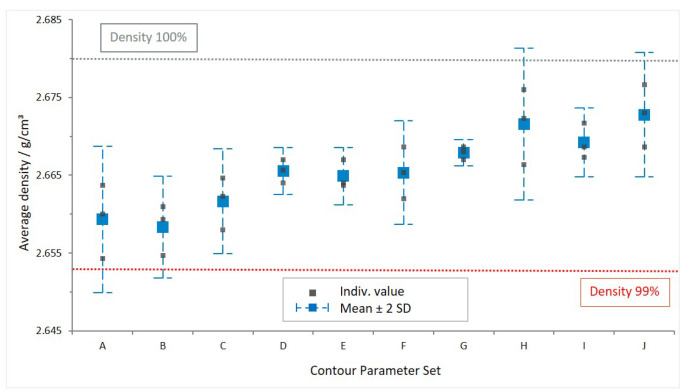
Density per manufacturing parameter set, mean ± 2SD; reference density: 100% = 2.68 g/cm^3^.

The data shown in Figure 6 confirm a density of over 99% for all of the measured samples, denoted by the grey squares in the graph, with a reference density of 2.68 g/cm^3^ (theoretical maximum).

When taking a 95% confidence interval (2SD), denoted by the dashed blue whiskers, into account, data sets A and B are below that 99% value. Data set A has a lower boundary value at 98.84%, which is also the lowest overall value.

From all data sets, the only statistically distinctive sets considering the depicted 95% confidence interval (2SD) are B and G. However, they cannot be distinguished from the remaining data sets by that requirement.

In Figure 6, an increasing tendency of density for higher scan speed (A—lowest scan speed to J—highest scan speed) is observed. Supposedly, this is caused by the occurrence of closed porosities that can not be filled with ethanol during weighing. Possibly, the close proximity of the cuboids on the build platform during production plays a role as well, as the trend cannot be observed in the density data of the mechanical testing samples included in Table A2.

However, the overall mean and %SD including all 90 measured values (3 samples each for 10 contour parameter sets, 3 measurements each) are 99.5% and 0.3%, respectively. The mean and %SD taking individual groups A to J into account amount to 99.5% and 0.17%, respectively.

In conclusion, the evaluated sample sets are considered comparable. It is found from the presented results that the bulk scan parameters predominantly define the part density. Thus, varying contour scan parameters has no statistically significant influence on the density.

## 4. Results and Discussion of Mechanical Characterization

### 4.1. Tensile Properties

**Figure 7:**
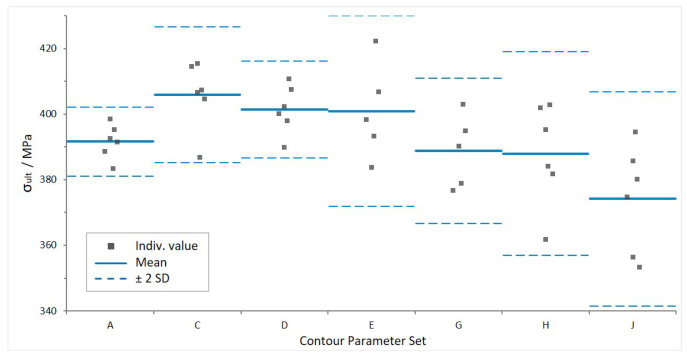
Ultimate tensile strength for seven different surface conditions, mean ± 2SD.

Figure 7 shows the tensile testing results for longitudinal specimens manufactured using the contour parameter sets A, C, D, E, G, H and J. Density values for the mechanical testing samples, along with all numerical data presented in Figure 7, are included in Appendix A.

Similar to the results from density determination, there is no statistically significant difference in UTS. The mean values of the individual surface conditions are between 374 and 406 MPa. For context, values reported in the literature for as-built of the same material and build direction vary from 300 MPa [49] to over 400 MPa [30,39,40]. For the cast alloy with T6 heat treatment, typically values of UTS between 320 and 360 MPa are reported [30]. Hence, the tested samples perform equally well or better than other as-built LPBF AlSi7Mg0.6 specimens and mostly exceed the strength of the cast material.

From the graph, it can be observed that the standard deviation increases for rougher surface textures. A possible influencing factor is the caliper cross-section measurement, since the UTS depends on the cross-sectional area. The caliper may be locked by protruding features, leading to variation in measured cross-section.

Moreover, the combination of line energy and powder application is a potential explanation. Poor flowability properties affect the homogeneity of powder dispersion within a layer. At higher contour scan speeds, the high reflectivity and fast heat dissipation may lead to irregular density of molten material, causing coarser surface texture and different microstructural properties. The latter will have to be confirmed by a microstructural analysis.

The overall SD of UTS values, including all 40 test results, is low—3.8% (14.94 MPa). The SD within each group (1.3% to 4.4%) is of the same order of magnitude as the SD between the groups (2.5% between mean values). Hence, the sample groups produced with different contour scan parameters are considered comparable with regard to tensile strength.

### 4.2. Fatigue Properties

**Figure 8:**
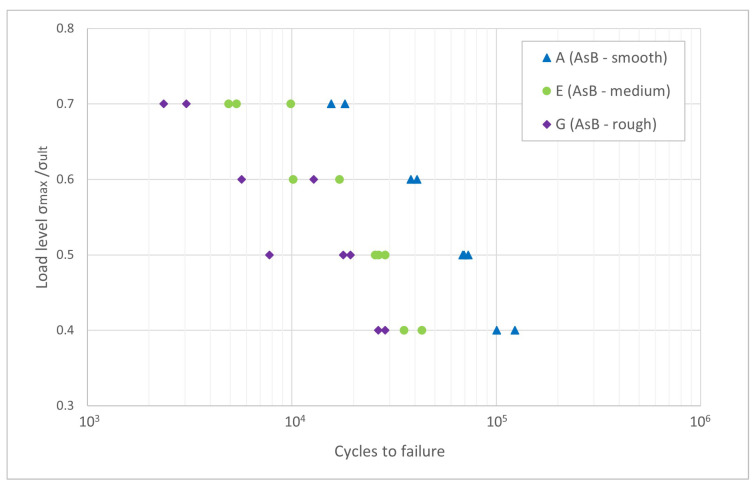
σ−N -curve for surface conditions A (AsB-smooth), E (AsB-medium) and G (AsB-rough), reference stress $\sigma_{ult}=\;392$ MPa. Reproduced from [52].

The fatigue testing results for surface conditions A, E and G for load levels $\sigma_{max}/\sigma_{ult}$ 0.4, 0.5, 0.6 and 0.7 are presented in Figure 8. The smoothest surface condition, A, has the best fatigue performance for all load levels and low scatter, as expected. Even for the highest tested load level, cycle numbers above $10^{4}$ are reached. Surface condition E exhibits some scattering for higher load levels, while condition G already shows scatter for load level 0.5 $\sigma_{ult}$ . A clear tendency towards higher fatigue resistance for smoother surfaces is visible. The same is reported in surface fatigue studies that include post-processing [7,10,15,43,45,54]. A possible explanation for the scatter on E and G is that, for these sample groups, the non-linear low cycle regime is reached. On a Wöhler curve, the logarithmic-linear relationship between stress and number of cycles is only valid in the high cycle fatigue regime [62]. Another reason may be the coarser surface texture caused by lower line energy and the possibly uneven powder distribution, as previously mentioned in Section 3.1 and Section 4.1.

The data in Figure 8 were previously published in [52], where the following sample naming was used: A—AsB-smooth, E—AsB-medium and G—AsB-rough. In [52], more detailed evaluations of surface texture and crack initiation are shown.

#### 4.2.1. Comparison with As-Built Surface Data from the Literature

To allow for comparison with surface quality data from the literature [39,46,51], Table 6 contains surface texture parameters generated with the respective cut-off values for one sample per surface condition.

Ra was determined from a 12 mm line profile, as indicated by Cacace et al. [46]. Sa and Sv were calculated from a 3 mm × 20 mm measured area. For measurement details, refer to [52]. A cut-off L-Filter of 0.8 mm was applied, as applied by Nasab et al. [51]. Please note that, deviating from Nasab et al., a least squares plane F-operation was used. The difference in F-operation is due to the sample geometries. This study assessed a flat sample geometry, while Nasab et al. evaluated cylindrical specimens.

Similarly to this paper, Nasab et al. [51] also used different as-built surfaces. However, taking a closer look at their considered surface conditions denoted S01, S05 and S07, they report larger Sv -values. Their best surface condition is S01 with Sv=112 µm, having the order of magnitude of the roughest surface considered in this study, G (AsB-rough). S05 with Sv=190 µm and S07 with Sv=205 µm largely exceed the values presented in Table 6. In their work, they induced defects to demonstrate their influence on rotating bending fatigue. They suggested a minimum material removal based on surface texture parameter results to improve surface quality. In contrast, this work was aimed at producing high-quality surfaces (described by Sa and Sk ) in as-built condition with no intention of surface post-processing.

Additionally, the samples investigated in this work have a smoother surface finish (see Table 6) in comparison with Cacace et al., who state an as-built Ra>10 µm. They sandblasted the samples to achieve an Ra<10 µm, as required for standard fatigue testing. The same holds for Grande et al., who reported an as-built Ra µm with 10<Ra<15 µm [39,46].

#### 4.2.2. Estimation of Stress at the Endurance Limit

The stress at the endurance limit was estimated based on the horizon method, using the logarithmic-linear equation

(1) lg(N)=m·lg(σ)+c

as described by Einbock [62]. For the sample groups A, E and G, the mean values for each load level were used to obtain their respective logarithmic-linear relations. The coefficients per surface condition are given in Table 7. For condition G, data points for load level 0.7 were in the LCF range with $N_{mean}\;=\;2.7\times 10^{3}$ . They are most likely not on the linear part of the S–N curve and were therefore excluded from this calculation.

**Figure 9:**
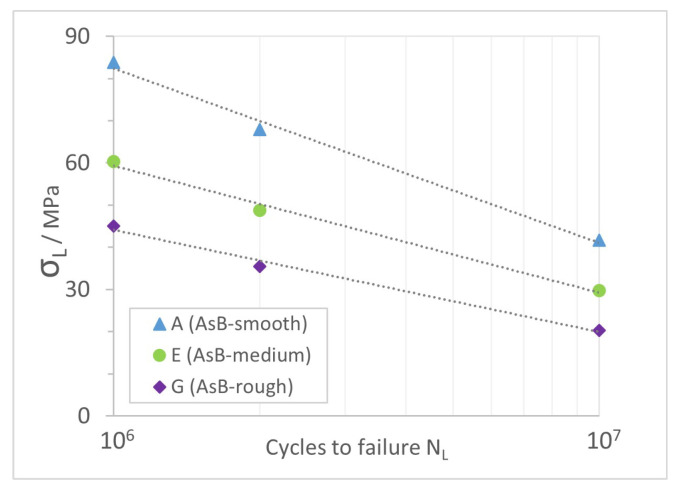
Estimated stress for different endurance limit values $N_{L}$ .

From this equation, the stress at endurance limit $\sigma_{L}$ was calculated for $N_{L1}\;=\;10^{6}$ , $N_{L2}\;=\;2\times 10^{6}$ and $N_{L3}=10^{7}$ and is presented in Table 8 and Figure 9. $N_{L1}$ to $N_{L3}$ were chosen to allow for comparison with literature values [39,43,45,46,49,63].

$\sigma_{L2}=49$ MPa for surface condition E corresponds well with the experimental findings of Denti and Sola [43] and Gatto et al. [45], who report mean values of 50 MPa for as-built specimens. Bassoli et al. [49] found a slightly higher $\sigma_{L2}$ of 60 MPa, which is in between groups A and E. However, as they did not evaluate surface quality, no direct comparison is possible.

Cacace et al. and Grande et al. [39,46] found experimental endurance limit stress values at $N_{L3}=10^{7}$ between 122 and 137 MPa for different positions on the build platform, being three times as high as found for A, the best performing condition studied here. As previously mentioned, they applied a sandblasting finish to meet the requirement for fatigue testing. Not only did this improve the surface finish, it also introduced compressive residual stresses, which prevent crack propagation [43,50].

Compared to the post-processed surface conditions presented by Denti and Sola [43] and Gatto et al. [45], the A condition’s endurance stress matches the performance of laser shot processed and metal shot peened (S70) specimens.

Considering conventionally manufactured parts, Dezecot and Brochu estimated a fatigue strength of 73 MPa for as-cast AlSi7Mg0.6 material from investment casting [63] at $N_{L}=10^{6}$ . Surface condition A exceeds this value by 15%. This increased strength is supposedly related to the fine microstructure due to faster solidification of the material in the LPBF process.

#### 4.2.3. Relationship of Surface Quality and Fatigue

In addition to the surface fatigue relationship shown in [52] for experimental values, this section presents data factorized to a load level of 0.5 $\sigma_{ult}$ .

**Figure 10:**
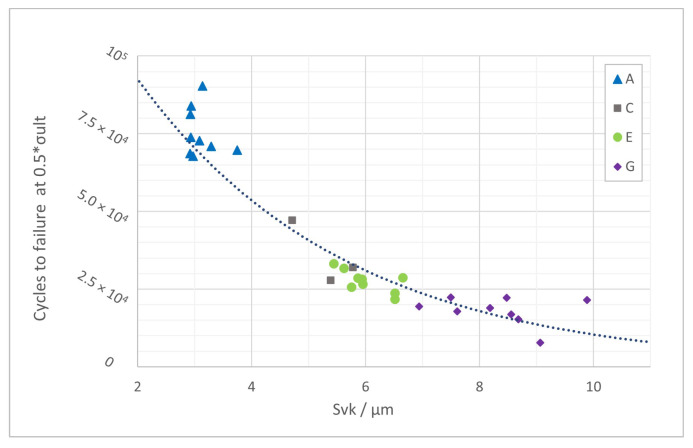
Exponential fit Svk vs. cycles to failure at 0.5 $\sigma_{ult}$ .

The factorization was done based on a linear regression across all data from the tested load levels. The exponential fit of Svk vs. the number of cycles to failure data in Figure 10 is described by

(2) $$N=159843e^{-0.273\cdot Svk}$$

with $R^{2}=0.8721$ . Numerical values for Svk are given in Table 9. Apart from the previously presented data for the A, E and G groups, there were also a few test results available from surface condition C samples, which were included in this fit.

The reduced valley depth Svk , derived from the material ratio curve, was chosen because it represents the valley population of a sample (within the measured area), as opposed to the common parameters for surface fatigue correlations Sz and Sv , which are individual extreme values and may not be representative of the considered surface.

This parameter choice is confirmed by the data shown in Table 6 and Table 8. Surface conditions E and G have comparable Sv but different Ra and Sa . As previously discussed, the fatigue life for both groups clearly differs as well.

Furthermore, especially when looking at rougher surface conditions, cracks tend to start from multiple locations at the surface. Among the tested samples, this was observed for all group G specimens and half of group E specimens (for details, refer to [52]). Hence, considering the specific nature of typical LPBF-processed surfaces, it makes sense to consider more than just one extreme value per surface.

## 5. Conclusions

This work aimed to produce samples with identical bulk and different surface quality, including high quality, to assess the impact of as-built surface texture on mechanical properties.

The evaluation of as-built surfaces was motivated by the desire to apply LPBF for complex geometries and inner surfaces, which may be complicated or infeasible to post-process. In addition, achieving the same surface finish and fatigue performance without post-processing saves time and resources.

The variation in surface texture was achieved by varying contour scan speed. The comparability of bulk quality for the different sample groups was confirmed by means of Archimedes’ density and tensile testing. The endurance limit was estimated based on four tested fatigue load levels. The relationship between the reduced valley depth Svk for the different surface quality groups was shown using data factorized to load level 0.5 $\sigma_{ult}$ .

 

The following main conclusions are derived from the presented work:

- Distinctive surface conditions with Sk (L-filter 0.25 mm) between 4 µm and 16 µm were produced.
- All tested specimens have a density > 99%; thus, the influence of contour scan parameters is considered insignificant regarding density.
- The ultimate tensile strength of 393 ± 9.98 MPa was found to be independent of contour scan variation.
- Optimized contour scan parameters result in as-built quality superior to some post-processed surfaces, enabling the reduction of processing steps and time.
- Condition A reaches a fatigue resistance of 84 MPa at $10^{6}$ cycles, exceeding values for as-cast and some surface post-processed literature results.
- The reduced valley depth Svk results in a good fit across the groups for the factorized surface fatigue relation. Therefore, Svk was found to be a suitable parameter to describe surface quality.

## Figures and Tables

**Table 1:** AlSi7Mg0.6 powder composition: mass fraction per alloying element.

Al	Si	Mg	Ti	Fe
93.13	6.15	0.6	0.09	0.05

**Table 2:** Bulk scan parameters.

Material	Layer Thickness	Hatch Distance	Scan Speed	Laser Power	Pre-Sinter
AlSi7Mg0.6	30 µm	0.12 mm	1000 mm/s	195 W	No

**Table 3:** Variation of contour scan parameters.

Material	Layer Thickness	Hatch Distance	Scan Speed	Laser Power	Pre-Sinter
			300 mm/s		
			600 mm/s		Yes
AlSi7Mg0.6	30 µm	0.12 mm	900 mm/s	195 W	
			1200 mm/s		No
			1800 mm/s		

**Table 4:** Naming of sample groups based on contour scan variation.

**Scan Speed in mm/s**	300	600	900	1200	1800
**Pre-Sinter**	A	C	E	G	I
**No Pre-Sinter**	B	D	F	H	J

**Table 5:** Load levels and stress values for $\sigma_{ult}=\;392$ MPa and R=0.1 [52].

Load Level $\sigma_{\max}/\sigma_{\mathrm{ult}}$	$\sigma_{\max}$ /MPa	$\sigma_{\min}$ /MPa	$\sigma_{\mathrm{mean}}$ /MPa
0.4	156.8	15.7	86.2
0.5	196.0	19.6	107.8
0.6	235.2	23.5	129.4
0.7	274.4	27.4	150.9

**Table 6:** Surface texture parameters for comparison with the literature, cut-off 0.8 mm. Evaluated for one specimen per surface condition.

Surface Condition	Ra /μm	Sa /μm	Sv /μm
A (AsB-smooth)	3.153	3.478	20.09
E (AsB-medium)	5.649	6.987	93.78
G (AsB-rough)	7.362	9.316	96.57

**Table 7:** Coefficients of logarithmic-linear equation lg(N)=m·lg(σ)+c for three surface conditions.

Coefficient	A (AsB-Smooth)	E (AsB-Medium)	G (AsB-Rough)
m	−3.292	−3.244	−2.892
c	3.794	3.364	3.283

**Table 8:** Estimated stress for different endurance limit values $N_{L}$ .

Surface Condition	A (AsB-Smooth)	E (AsB-Medium)	G (AsB-Rough)
$\sigma_{L1}$ at $N_{L1}=10^{6}$ /MPa	84	60	45
$\sigma_{L2}$ at $N_{L2}=2\times 10^{6}$ /MPa	68	49	35
$\sigma_{L3}$ at $N_{L3}=10^{7}$ /MPa	42	30	20

**Table 9:** Svk for fatigue-tested samples.

Surface Condition	Mean Svk/ µm	SD Svk/ µm
A	3.105	0.271
C	5.296	0.539
E	6.028	0.431
G	8.321	0.890

## Data Availability

Data are available from the corresponding author upon request.

## Appendix A

**Table A1:** Sa and Sk for cuboid samples, 10 surface conditions L-filter 0.25 mm, S-filter 20 µm. With pre-sinter (right), without pre-sinter (left).

Parameter Set	J	H	F	D	B	A	C	E	G	I
Scan Speed/mm/s	1800	1200	900	600	300	300	600	900	1200	1800
Sa /µm, Mean (N = 3)	5.94	3.88	2.33	1.82	1.54	1.42	1.63	2.17	3.61	5.85
Sa /µm, SD	0.10	0.34	0.13	0.08	0.06	0.08	0.04	0.07	0.15	0.61
Sk /µm, Mean (N = 3)	15.93	10.25	6.66	5.35	4.53	4.30	4.88	6.49	10.50	16.09
Sk /µm, SD	0.26	0.67	0.24	0.15	0.05	0.18	0.15	0.06	0.37	1.18

**Table A2:** Ultimate tensile strength and relative density of tensile samples. Reference density 2.68 g/cm^3^. Seven surface conditions.

Parameter Set	A	C	D	E	G	H	J
N	6	6	6	6	6	5	5
$\sigma_{ult}$ /MPa, Mean	391.63	405.90	401.42	400.87	388.79	387.96	374.21
$\sigma_{ult}$ /MPa, SD	5.26	10.33	7.37	14.54	11.04	15.55	16.33
Relative Density/%, Mean	99.85	99.68	99.52	99.61	99.69	99.58	99.74
Relative Density/%, SD	0.17	0.15	0.18	0.11	0.17	0.28	0.20
